# *In Vivo* Quantitative Microcomputed Tomographic Analysis of Vasculature and Organs in a Normal and Diseased Mouse Model

**DOI:** 10.1371/journal.pone.0150085

**Published:** 2016-02-24

**Authors:** Nanditha Mohan Das, Sarah Hatsell, Kalyan Nannuru, Lily Huang, Xialing Wen, Lili Wang, Li-Hsien Wang, Vincent Idone, Jeffrey A. Meganck, Andrew Murphy, Aris Economides, LiQin Xie

**Affiliations:** 1 Department of Skeletal Diseases – Therapeutic Focus Areas, Regeneron Pharmaceuticals Inc., Tarrytown, New York, United States of America; 2 Research and Development, PerkinElmer, Hopkinton, Massachusetts, United States of America; MD Anderson Cancer Center, UNITED STATES

## Abstract

Non-bone *in vivo* micro-CT imaging has many potential applications for preclinical evaluation. Specifically, the *in vivo* quantification of changes in the vascular network and organ morphology in small animals, associated with the emergence and progression of diseases like bone fracture, inflammation and cancer, would be critical to the development and evaluation of new therapies for the same. However, there are few published papers describing the *in vivo* vascular imaging in small animals, due to technical challenges, such as low image quality and low vessel contrast in surrounding tissues. These studies have primarily focused on lung, cardiovascular and brain imaging. In vivo vascular imaging of mouse hind limbs has not been reported. We have developed an *in vivo* CT imaging technique to visualize and quantify vasculature and organ structure in disease models, with the goal of improved quality images. With 1–2 minutes scanning by a high speed *in vivo* micro-CT scanner (Quantum CT), and injection of a highly efficient contrast agent (Exitron nano 12000), vasculature and organ structure were semi-automatically segmented and quantified via image analysis software (Analyze). Vessels of the head and hind limbs, and organs like the heart, liver, kidneys and spleen were visualized and segmented from density maps. In a mouse model of bone metastasis, neoangiogenesis was observed, and associated changes to vessel morphology were computed, along with associated enlargement of the spleen. The *in vivo* CT image quality, voxel size down to 20 μm, is sufficient to visualize and quantify mouse vascular morphology. With this technique, *in vivo* vascular monitoring becomes feasible for the preclinical evaluation of small animal disease models.

## Introduction

Changes in the vascular network and organ morphology in the body have been associated with the emergence and progression of many diseases, including bone fracture, inflammation and cancer [1–3]. Specifically, angiogenesis has been shown to play a vital role in the development of tumors and the subsequent spread of cancer. Tumor blood vessels and normal blood vessels have been identified as differing in network structure and blood flow, with the former characterized by a circuitous framework and slower circulation. Metastatic bone tumors have also been found to be highly vascularized, with vessels having similar attributes of shape and organization as other tumor blood vessels, as well as being enlarged in diameter [4]. Thus, high quality *in vivo* visualization and quantification of vascular changes in small animal models of human disease, especially bone metastases, may aid the detection and understanding of disease pathogenesis, and the evaluation and preclinical development of novel drug treatments for inflammation and tumor growth in the same [5]. Such investigation of vascular networks and organ morphology facilitates preclinical, longitudinal research.

Many imaging techniques have been employed to help visualize vascular networks and organ morphology in murine models of disease, and micro-computed tomography is evolving as a powerful tool for imaging both *ex vivo* and *in vivo* [6]. Both have applications in the monitoring of angiogenesis in bone fracture healing, osteoarthritis, and tumorigenesis [3, 7, 8]. Currently, *ex vivo* micro-CT is able to generate higher resolution images of vasculature than *in vivo* micro-CT [9, 10]. However, as long as radiation dose can be properly managed, *in vivo* imaging offers unique advantages to the study of angiogenesis that *ex vivo* cannot—it would enable the non-invasive, longitudinal monitoring of animal models of disease, as well as provide quantitative analysis of the effectiveness of therapeutic agents targeting disease [11], while also requiring the use of fewer experimental animals [12].

The x-rays of the micro-CT only allow for the examination of density contrast with respect to the surrounding environment, like the lungs, fat and bones. Soft tissues are less dense and often have similar densities or contrast to the surrounding tissues, leading to difficulties in differentiating between them [13]. To increase the contrast between different soft tissues, contrast agents have been developed and their differential distribution can be leveraged to help discern other organs and vessels within experimental mouse disease models. However, clinical contrast agents, namely hydrophilic iodinated molecules with low molecular weights, have not necessarily been ideal for vascular imaging in mice. This is because mice have a physiological heart rate between 500–620 bpm [14]. Although the heart rate can slow to 343–428 bpm when anesthetized [15], the agent Hypaque-M still has a peak contrast approximately 10 seconds post-injection [16]. Similarly, Isovist 300 clears so quickly that vascular imaging is difficult [17]. Although the latest generation of commercially available scanners is fast enough to image these kinetics, in practice the time associated with animal handling and scan initiation often necessitate continuous perfusion of iodinated agents for vascular imaging.

The best imaging scenario involves a contrast agent that is able to achieve a relatively long-lasting contrast, while minimizing injection volume burden to the experimental animals and reducing scanning time [18–20]. Several contrast agents, with slower kinetics, have been developed and used to produce images of organs that were not previously isolated [18, 21]. These agents with slower kinetics are easier to use for vascular imaging [17]. Exitron nano 6000 and Exitron nano 12000 (Viscover ExiTron nano; Miltenyi Biotec, Bergisch-Gladbach, Germany) are alkaline earth metal—based nanoparticulate contrast agents, with a mean hydrodynamic particle diameter of 110 nm. Exitron nano is taken up by, and remains in, the reticuloendothelial system (RES) of macrophages. These agents have been designed for particularly strong and long-lasting contrast of blood vessels to optimize imaging windows and scan time [18, 21].

Despite advancements in imaging technology, there are only a few published papers describing the *in vivo* imaging of vasculature in small animals [10, 13, 20, 22–24]. These studies have primarily focused on lung, cardiovascular, and brain imaging, as well as tumor angiogenesis (lung, brain, mammary, skin). The quality of the images obtained from the *in vivo* studies has not been sufficient for the monitoring of the vascular network. Image resolution is typically limited to only about 50 μm for vessels [10], making the visualization of smaller vessels in a disease model difficult [25], especially in a mouse model. Mouse limbs have been widely used in preclinical studies, such as bone fracture healing and bone metastasis. Prior studies of bone metastasis have examined Osteolysis using *in vivo* μCT [26, 27], and examined rat vasculature using *in vivo* [28] and mouse vasculature using *ex vivo* μCT [4]. However, *in vivo* vascular imaging in the hind limbs of a mouse model has not been reported.

The aim of this study was to develop an *in vivo* CT imaging technique, with high image quality, to monitor vascular changes in a preclinical mouse model. We were able to develop high quality segmented images and density maps of vasculature and organ morphology using a higher dose of ExiTron nano 12000, which was well tolerated by the mice, without any changes to eating behavior or mobility. We also monitored the progression of bone cancer with time by visualizing an increase in blood vessel growth (angiogenesis) in the mouse hind limbs, quantifying associated vascular morphological changes, and observing a dramatic increase in the size of the spleen.

## Methods and Materials

### Ethics Statement and Animal Preparation

All the mice used in this study were housed under specific pathogen free environment at the Regeneron Pharmaceuticals Inc. animal research facility. Autoclaved water and sterile mouse chow were provided ad libitum. All experimental protocols, anesthesia, surgical procedures, tumor implantation procedures and analgesic use to alleviate pain used in this study were approved by the Regeneron Institutional Animal Care and Use Committee. Mice were provided with enrichment during the course of the study including nestlets made of cotton fibers for shredding and nesting behavior. All efforts were made to minimize suffering during the study procedure.

### Contrast Agent

For this particular study, only one contrast agent was used: ExiTron^™^ nano 12000 (Viscover^™^), purchased from Miltenyi Biotec GmbH (Bergisch Gladbach, Germany). ExiTron™ nano 12000 is a high density, alkaline earth metal-based nanoparticulate contrast agent specifically formulated for pre-clinical computed tomography. These nanoparticles have a mean hydrodynamic diameter of 110 nm. When ExiTron^™^ nano 12000 is intravenously injected into mice, it circulates in the blood stream for a prolonged time. Because the nanoparticles are too large to be excreted by the kidney, they accumulate in the liver (in Kupffer cells) and spleen due to uptake by macrophages (by the reticuloendothelial system), where they stay for months, facilitating longitudinal studies [21].

The entire volume of ExiTron^™^ nano 12000 (250–300 μl) was injected into the tail vein of each 12 week old female Balb/C mouse (takes around 10 seconds). The recommended dosage by the manufacturer is 150 μl/25 g mouse [12]. ExiTron^™^ nano 12000 circulates in the blood pool and takes 24 hours to clear in healthy mice [29]. The peak contrast occurs at around 4 hours [13, 21].

### μCT Imaging (Setup and Analysis)

CT imaging was performed using a high speed *in vivo* μCT scanner (Quantum FX, PerkinElmer, Hopkinton, MA, USA). The X-ray source was set to a current of 160 μA, voltage of 90 kVp. The CT imaging was visualized via 3D Viewer, existing software within the Quantum FX system. The field of view (FOV) was 10 mm x 10 mm, and voxel size was 20 μm. Initially, a larger FOV was employed and then subsequently narrowed to see the effect on the resulting resolution, to identify the best possible imaging parameters for the various anatomical locations of interest (i.e. head, hind limbs, etc.).

The animals received light anesthesia to immobilize them during scanning. Specifically, they were anesthetized with isoflurane (2.5–3% to minimize motion artifacts from respiration and heart beats) and then positioned on the scan platform. Constant delivery of isoflurane was achieved via a nose cone connected to the scan platform. The operator initiated the scan from a nearby computer terminal, and the scanning process typically lasted less than 5 minutes (2–4 minutes per mouse). Following the scanning process, mice were revived under a heating lamp and returned to their home cages.

### Image Processing

Following scanning, image processing steps were undertaken. Image segmentation was performed semi-automatically using the Volume Edit tools within the Analyze software package (AnalyzeDirect, Overland Park, KS, USA). Briefly, segmentation masks (object maps) were created using a combination of semi-automatic and manual techniques (object extraction, region growing and thresholding tools). These segmentation results were then manually modified if necessary and quantified using the ROI tools.

Data was first loaded into the Analyze Direct software. The ‘Volume Edit’ module was opened, and the ‘Semi-automatic’ tab was selected. ‘Walls’ were drawn to define the region of interest (ROI). ‘Region Grow’ or ‘Object Extractor’ was selected, and a point seeded. Vessels are segmented mainly by ‘Region Grow’ and organs by ‘Object Extractor.’ The upper and lower limits of the threshold range were adjusted manually so their respective values were descriptive of the organs and vasculature under study. The threshold is organ-specific. The object was then grown/extracted. The module applies thresholding, morphology erosion and dilation, and region growing steps in an attempt to automatically segment an object within a volume. These steps can be cyclic if multiple organs (systems) are being segmented. Within the ‘Semi-automatic’ tab, there is also the option to use the ‘Object Separator,’ in which two points are seeded, the first becoming the new object, and the second remaining the old object. Separation can also be done manually. Finally a 3D rendering of the organs and vasculature can be generated, which can be saved as an object map from which volumes can be calculated using the ‘ROI’ module. A schematic of the image processing steps is demonstrated in S1 Fig.

### Intratibial Implantation Model of Tumor Induced Osteolysis (Murine Model of Bone Metastasis)

An osteolytic bone metastasis model (Intratibial Tumor Implantation) was used to phenocopy the human bone metastasis condition, where patients suffer tumor induced bone destruction during bone metastasis. The model was used to investigate new therapeutic drugs to inhibit tumor-induced osteolysis, and to determine angiogenic changes during osteolytic bone metastasis [30, 31].

Eight-week-old female Balb/C mice (The Jackson laboratory, Bar Harbor, Maine) (n = 2, each, for control and experimental groups) were anaesthetized using 120 mg/kg ketamine and 50 mg/kg xylazine i.p. The implantation site, right knee joint was shaved and sterilized with isopropyl alcohol, cleaned with Betadine, and allowed to dry. A small incision was made on the skin and fascia, lateral to the knee joint and gluteus. The quadriceps muscles were separated at the joint using blunt forceps, and the whole kneecap was pushed towards the medial side to reveal proximal tibial tuberosity. Using the ridge between the femur distal condyles as guidance, a small hole was drilled into the tibial plateau with a 25-gauge needle. X-ray radiographs were taken immediately to confirm the correct location of implantation. Then, a Hamilton syringe (50 μl capacity, 26S gauge) was inserted in the proximal end of the tibia, and 2.5 X 10^4^ 4T1-luc tumor cells suspended in 20 μl PBS were injected into the intramedullary space. Mice were given two doses of analgesic Buprenex (0.1 mg/kg). During the course of the study, mice were monitored for overall health three times a week. In order to alleviate pain, mice were treated with Buprenorphine at 0.09 mg/kg body weight.

The progression of angiogenesis was monitored at two time points. Mice were injected with the contrast agent first at 2 weeks and then scanned after 2 hours (FOV: 10 mm x 10 mm). They were then rescanned the following day, where the liver and spleen could still be imaged. The mice were then injected again at 5 weeks with the contrast agent and subsequently scanned.

Since an orthotopic mouse model was used, where tumor burden cannot be measured externally, the health statuses of the mice were monitored via observation. Mice implanted with cancer cells were carefully monitored throughout the course of the study for signs of pain or distress, and loss of body weight. Tumor induced osteolytic changes were monitored by weekly radiography and risk of fracture was assessed. Any mouse exhibiting cachexia, rough coat, or distress, were immediately sacrificed. Despite the tumor burden, mice did not exhibit any serious adverse effects. Despite the osteolytic changes observed in the tumor-implanted mice, they exhibited gait and normal feeding behavior. As mentioned previously, any mice that were moribund or exhibiting any of the above-mentioned signs were immediately sacrificed (euthanasia via CO_2_ inhalation).

## Results

All animals tolerated the injection of 250–300 μl of ExiTron nano 12000 and no immediate behavioral changes were observed: body weight, food and water consumption, and movement remained the same as prior to contrast agent administration. Mice were administered the contrast agent no more than two times per animal. They were followed for between 5 and 6 months post contrast agent administration, and appeared to tolerate the contrast agent well, with no side effects noted. The spleen and liver did show dense concentration of the contrast agent when these mice were imaged with microCT. After 6 months, the Regeneron Pharmaceuticals IACUC required the sacrifice of these mice due to old age.

### Administration of Contrast Agent

**Fig 1:**
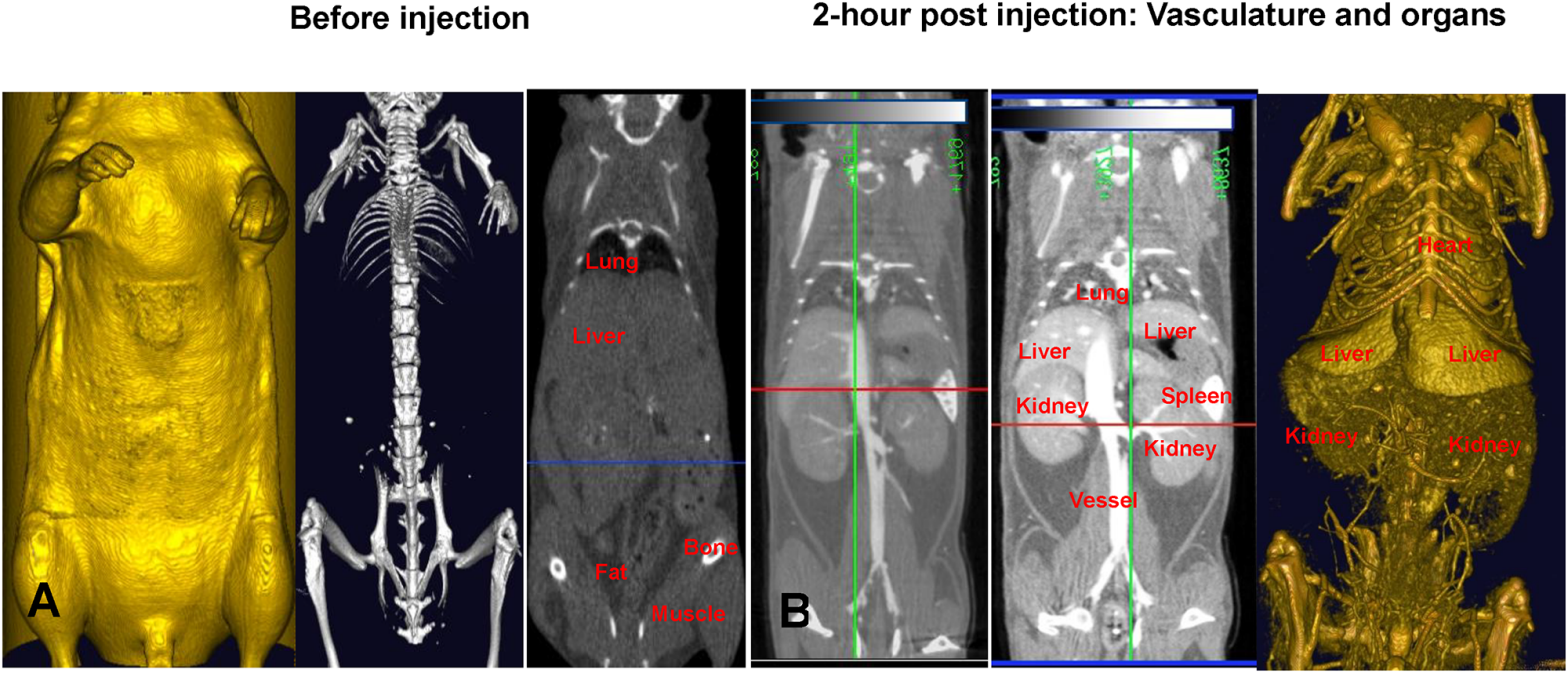
Visualizing the vasculature and organ structures in a live mouse using Exitron^™^ nano 12000 (FOV: 60mm×60mm; 2 min scan). The colored lines represent the orientation (plane) and specific location where a particular cross-sectional slice has been taken. The green line represents the sagittal plane, the red line represents the transverse plane, and the blue line represents the coronal plane. (A) Images in all three panels were taken before the injection of Exitron™ nano 12000. The panel on the left shows a 3D volume-rendered image of the body of the mouse. The middle panel shows a 3D volume-rendered image of the bone structure. The right panel is a density map showing the lungs, fat and bones (coronal plane). (B) Images in all three panels were taken 2 hours post injection. The left panel is a density map showing the liver, kidney, spleen, and vessels in a mouse administered 150 μl/ 25 g. The middle panel is a density map showing the liver, kidney, spleen and vessels in a mouse administered 250–300 μl/ 25 g. The right panel is a 3D volume-rendered image using the density map from the middle panel (300 μl/ 25 g dosing).

Before injection of the contrast agent, only bone, lungs and fat were visible with micro-CT imaging (Fig 1A). This is because these structures have different densities when compared to the surrounding structures. Organs like the liver and kidneys cannot be distinguished from each other because they are too similar in their densities and the densities of their surroundings. However, 2 hours after the injection of ExiTron nano 12000, organs (like the liver, kidneys, and spleen) and structures (large vessels) became more easily visible (Fig 1B). The left panel of Fig 1B depicts the contrast obtained with the recommended dose of ExiTron nano 12000 (150 μl/ 25 g mouse), whereas the middle panel shows the enhanced contrast and improved signal-to-noise ratio (SNR) obtained with a dose of 250–300 μl/ 25 g mouse. The boundaries of organs are discernibly sharper and clearer in the higher dose images, enabling more accurate segmentation during image processing. In addition, details of smaller vessels are better noted with the higher dose. The right panel shows a 3D volume-rendered image using the density map from the 300 μl/ 25 g dosing of ExiTron nano 12000.

### Visualization, Segmentation and Quantification of Vasculature & Organs

**Fig 2:**
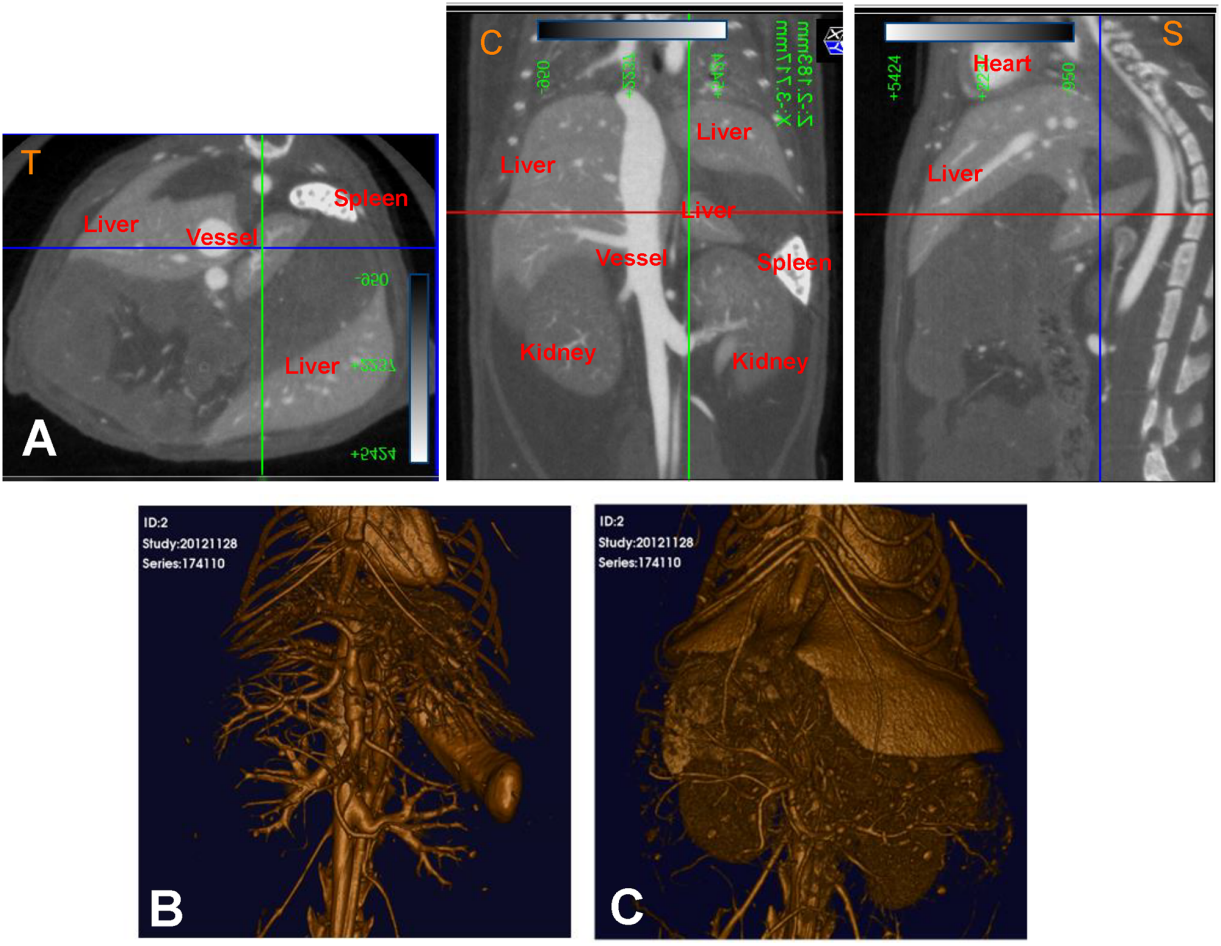
Adjusting the threshold allowed for the imaging of several vasculature and organ structures in a live mouse (FOV: 30mm×30mm; 2 min scan). The colored lines represent the orientation (plane) and specific location where a particular cross-sectional slice has been taken. The green line represents the sagittal plane, the red line represents the transverse plane, and the blue line represents the coronal plane. (A) A 3D volume-rendered image at a high threshold (350 Hounsfield Units (HU)) allowed visualization of the main vessel, bone, and spleen. (B) A 3D volume-rendered image at a low threshold (150 Hounsfield Units (HU)) allowed visualization of small vessels, liver, and kidneys. (C) The density maps used to create the 3D volume-rendered images (left to right—transverse, coronal and sagittal planes).

The vascular networks of different organs are diverse, attributed to the structural diversity of the endothelial cells that they are composed of [32]. The differential distribution of the contrast agent is thus possible, and enables organs to be detected as having distinct densities from the surrounding tissue by the x-rays of the microCT. Specifically, the circulating blood distributes the contrast agent and creates localized concentrated regions, which can be leveraged to capture a density map of the mouse organs (Fig 2C), and can be further used to generate 3D volume-rendered images and movies (Fig 2A and 2B; S1 and S2 Movies) (3D Viewer software, Quantum *in vivo* μCT). The main vessel and spleen have high density, and can thus be visualized and segmented using a high threshold (Fig 2A and S1 Movie). The liver has a low density, and the kidneys even lower, and can thus be visualized with lower thresholds (Fig 2B and S2 Movie).

**Fig 3:**
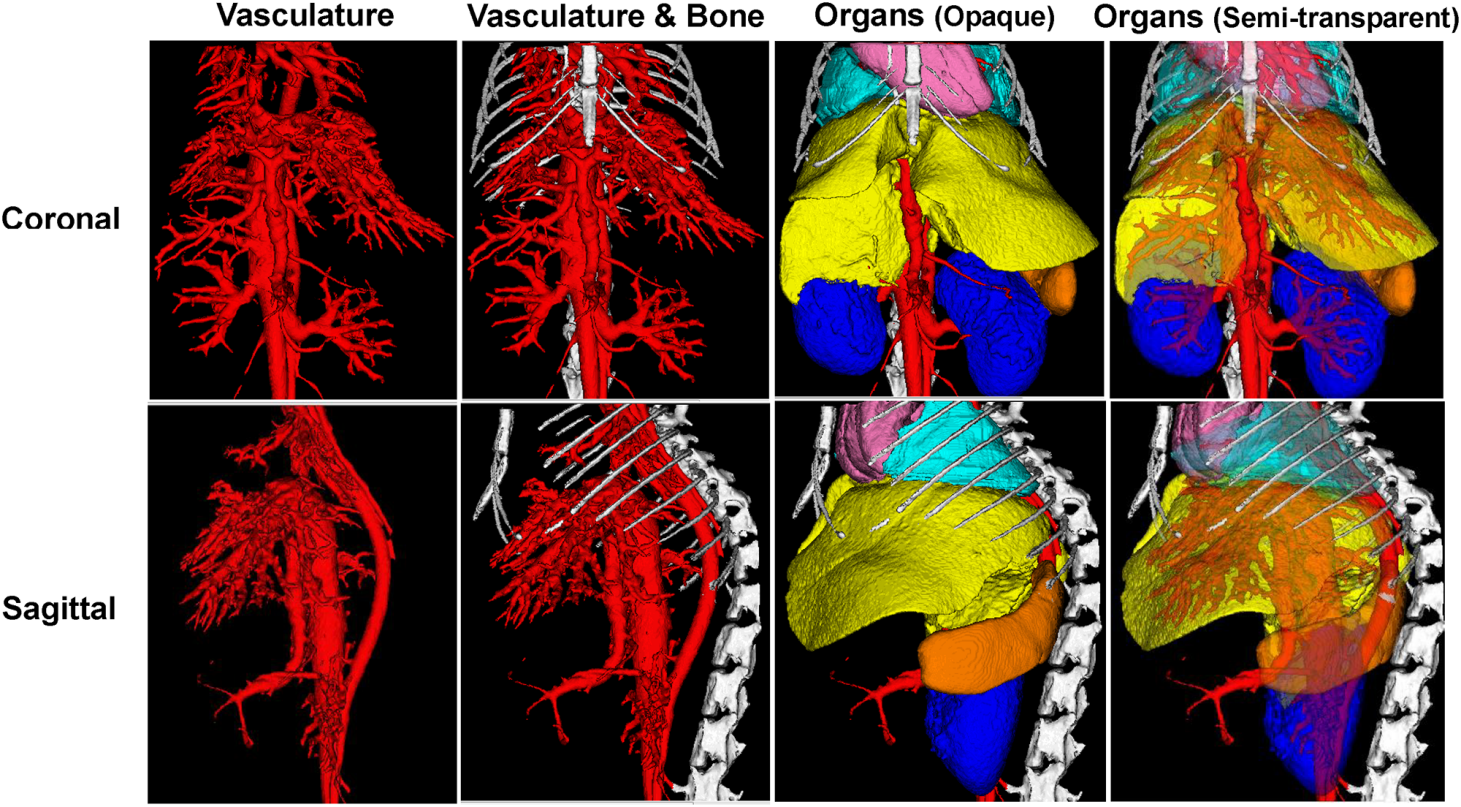
Organ volume, morphology and vessel distribution were quantified using *in vivo* μCT. Top row: The coronal view of the various vessels and organs in the mouse. Bottom row: The sagittal view of the various vessels and organs in the mouse. (FOV: 30mm×30mm; 2 min scan).

**Fig 4:**
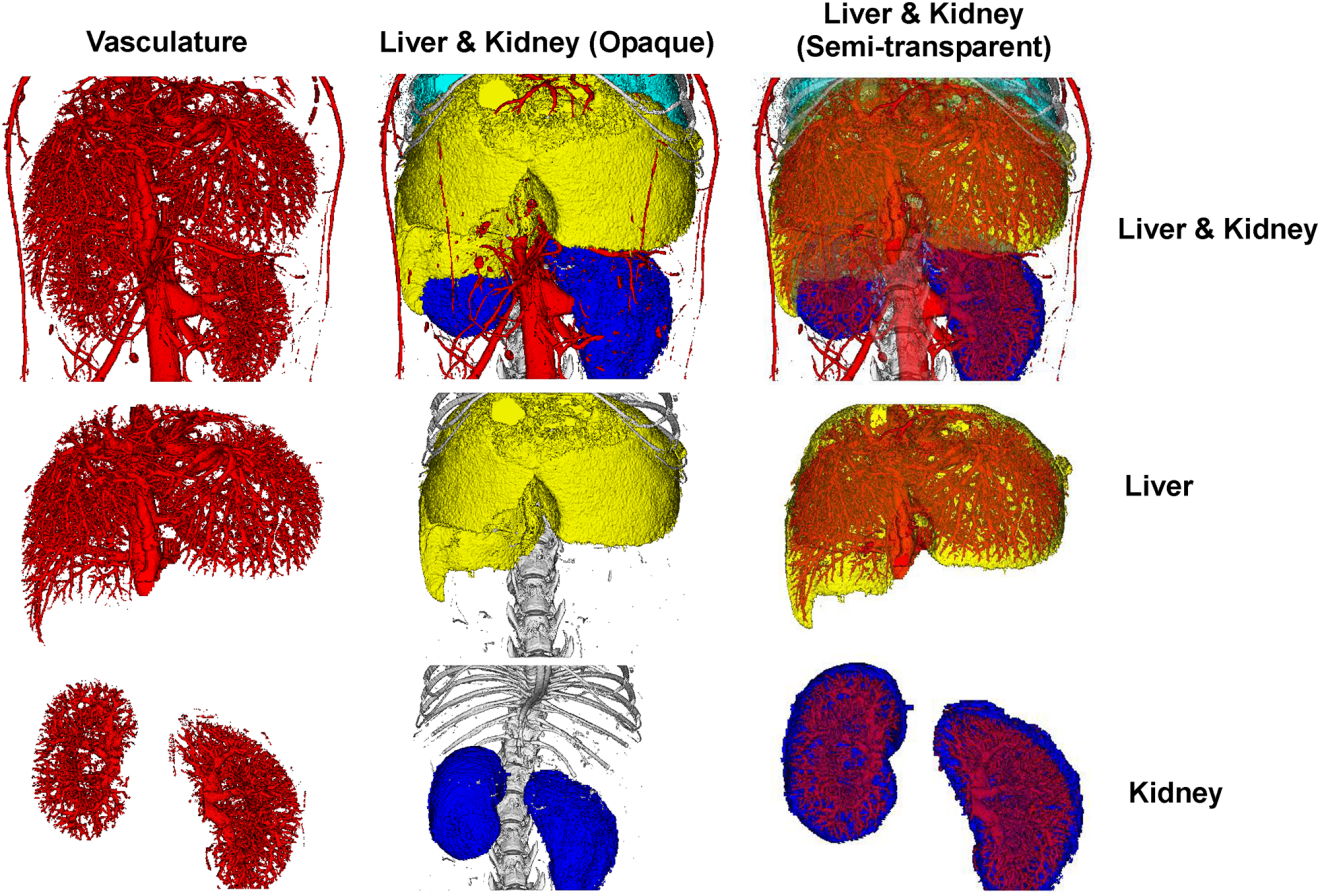
Vasculature in individual organs were quantified (FOV: 20mm×20mm; 4 min scan). The liver and the the kidneys, were viewed along with their vasculature. Top row: 3D volume-rendered images of the liver and kidney. Middle row: 3D volume-rendered images of just the liver. Bottom row: 3D volume-rendered images of just the kidney.

The software Analyze was utilized to segment and visualize organ morphology and vessel distribution in specific organs within the mouse (Fig 3). Segmentation was performed sequentially and hierarchically—the main vessel, as well as contiguous networks of blood vessels, adjacent bone, organs (S3 Movie), and finally semitransparent vasculature was superimposed on the organs (S4 Movie). Specifically, we focused on the morphology and the vascular systems of the liver and kidneys (Fig 4). Similar to Fig 3, vasculature was segmented, followed by the liver and kidneys, and then a semi-transparent depiction of the vasculature of the liver and kidneys were accomplished. Utilizing this method, organ volume and percent vascular volume were quantified for the kidneys and liver, shown in Table 1. In this way, abnormalities, if present in organs and vasculature, can be identified, in animal disease models, and the progression of these abnormalities can be monitored longitudinally.

**Table 1:** Organ Volume and Percent Vascular Volume.

Organ	Organ Volume^c^ (mm^3^)	Vascular Volume^d^ (mm^3^)	Total Volume^e^ (mm^3^)	% Vascular Volume^f^
**Kidney**^a^	198.17 ± 21.72	16.23 ± 2.04	214.40 ± 23.15	7.58 ± 0.66
**Liver**^b^	1160.64 ± 57.47	135.00 ± 14.06	1295.64 ± 52.93	10.44 ± 1.23

^a^ n = 6

^b^ n = 3

^c^ volume of the organ, not including any vasculature

^d^ volume of the vasculature alone

^e^ volume of the vasculature and organ together

^f^ percent volume of vasculature, obtained by dividing the vascular volume by the total volume and multiplying by 100

**Fig 5:**
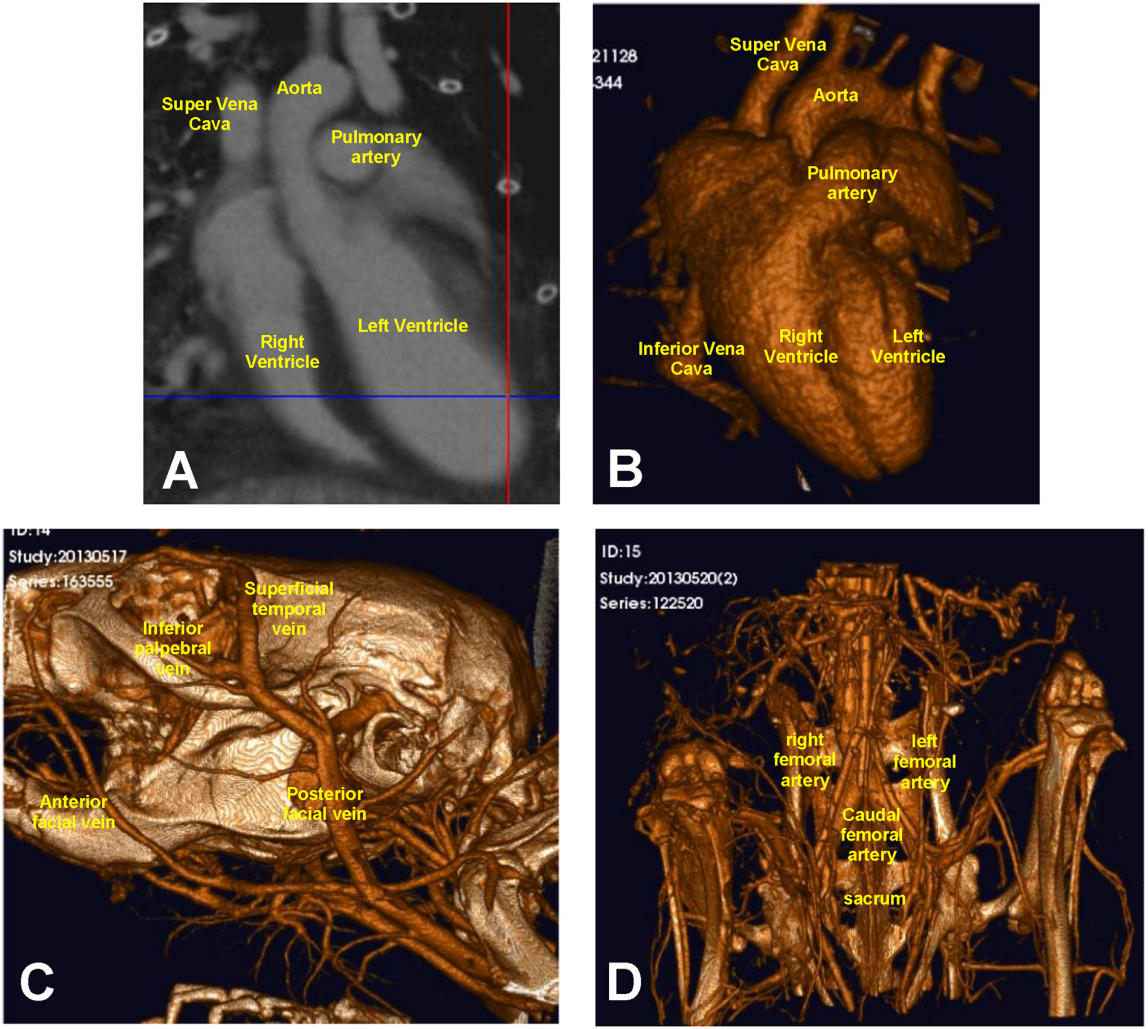
Vasculature can be imaged down to a 20 μm voxel size. (A) Density map of a mouse heart (FOV: 30mm×30mm; 2 min scan). (B) 3D volume-rendered image of a mouse heart (FOV: 30mm×30mm; 2 min scan). (C) 3D volume-rendered image of vasculature surrounding mouse head structures. The superficial temporal vein and posterior facial vein can be viewed in great detail in these images (FOV: 20mm×20mm; 2 min scan). (D) 3D volume-rendered image of vasculature surrounding mouse hip/hind limbs, including the left and right femoral artieries and caudal femoral artery (FOV: 20mm×20mm; 2 min scan).

Another organ of focus was the heart. Following the generation of a density map of a mouse heart (Fig 5A), a 3D volume-rendered image was created (Fig 5B). The 3D structure of the heart was visualized, with depictions of the aorta, super vena cava, the pulmonary artery, inferior vena cava, and right and left ventricles.

Reconstructed images were also employed to visualize vessels around the head and hind limbs of the mouse (Fig 5C and 5D and S5 and S6 Movies). Fig 5C and S5 Movie depict the skull and its associated network of blood vessels, including the posterior facial vein, superficial temporal vein, inferior palpebral vein andanterior facial vein. Fig 5D and S6 Movie depict the sacrum and hind limbs, and its network of blood vessels, including the right and left femoral arteries, and caudal femoral artery.

### Disease Monitoring

**Fig 6:**
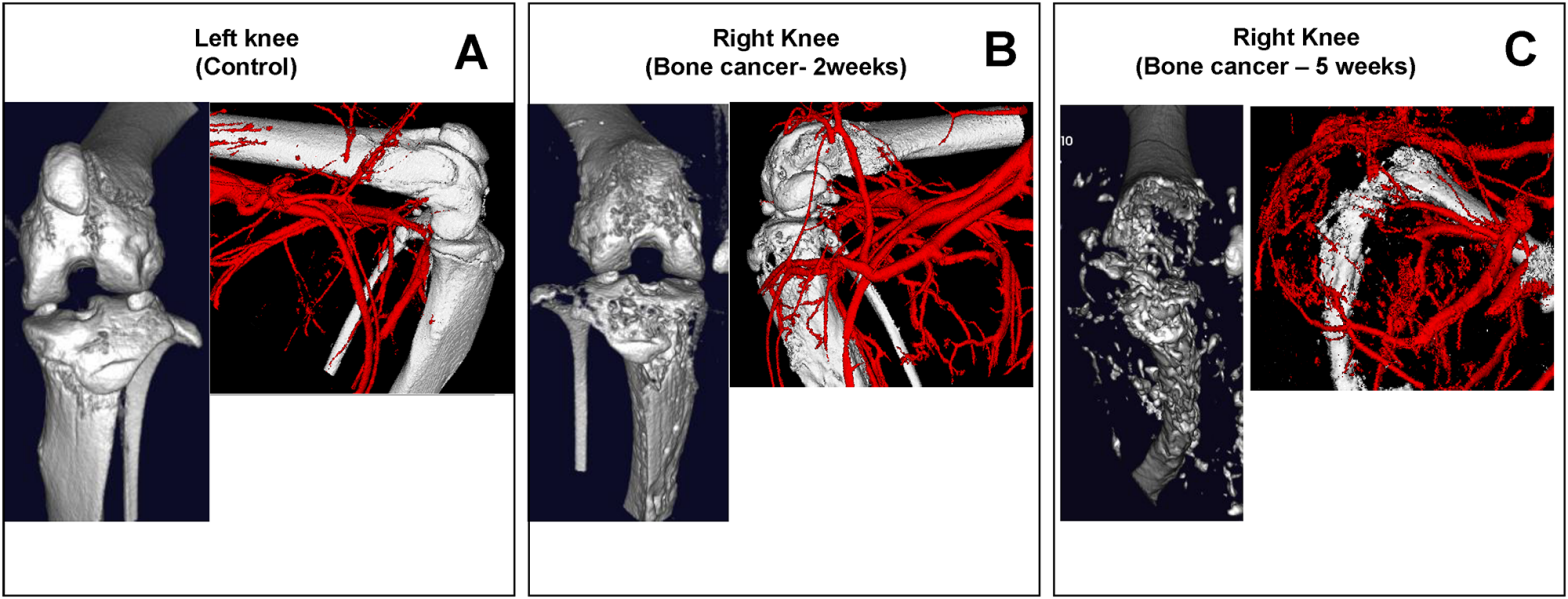
Angiogenesis can be monitored by using 3D imaging in a live mouse hind limb (FOV: 10mm×10mm; 2 min scan). A mouse bone metastasis model was used for these images (See Methods and Materials section for details). (A) The left knee served as a control (no cancer agent injected into the tibia); no angiogenesis was observed on the left knee at the tibial surface and little to no bone erosion was observed. (B) Right knee 2 weeks after cancer agent was injected into the tibia; angiogenesis was observed on the tibial surface of the knee and significant bone erosion is observed. (C) Right knee 5 weeks after cancer agent was injected into the tibia; an increase in angiogenesis is observed all over the knee and bone has mostly deteriorated.

Using this imaging method, the progression and emergence of diseases (in knock-out mice and other disease models) can be longitudinally monitored, due to the high quality images that can now be constructed. For example, we were able to observe angiogenesis in our bone metastasis mouse model (Fig 6). This first instance of 3D angiogenesis imaging in a live mouse hind limb enabled us to perceive the marked increase in the proliferation of vessels alongside the concurrent deterioration of bone, with the progression of the bone cancer. Fig 6A depicts a control knee joint with its system of blood vessels, and without any osteolysis. There is no vascularization at the tibial surface. Fig 6B shows a knee joint 2 weeks after induction of bone cancer. Osteolysis and increased vascularization from the norm (control) can be identified at the tibial surface. In addition to the growth of blood vessels, an apparent higher blood vessel volume (10.49 ± 0.63 mm^3^, n = 2) was observed in the metastatic right knee compared to a vessel volume in the control knee (5.38 ± 0.38 mm^3^) (Fig 6B). Fig 6C shows a knee joint at 5 weeks after the induction of bone cancer. Here, severe osteolysis has destroyed the joint and intense vascularization can be seen. Additionally, the blood vessels in the metastatic limb appear to have proliferated in disarray from 2 weeks to 5 weeks (Fig 6B and 6C), with vessel volume increasing to 15.07 ± 1.90 mm^3^. Vascularization corresponded to osteolysis and tumor growth, occurring in regions of severe bone degeneration, and forming a tortuous architecture that completely surrounds the tumor.

**Fig 7:**
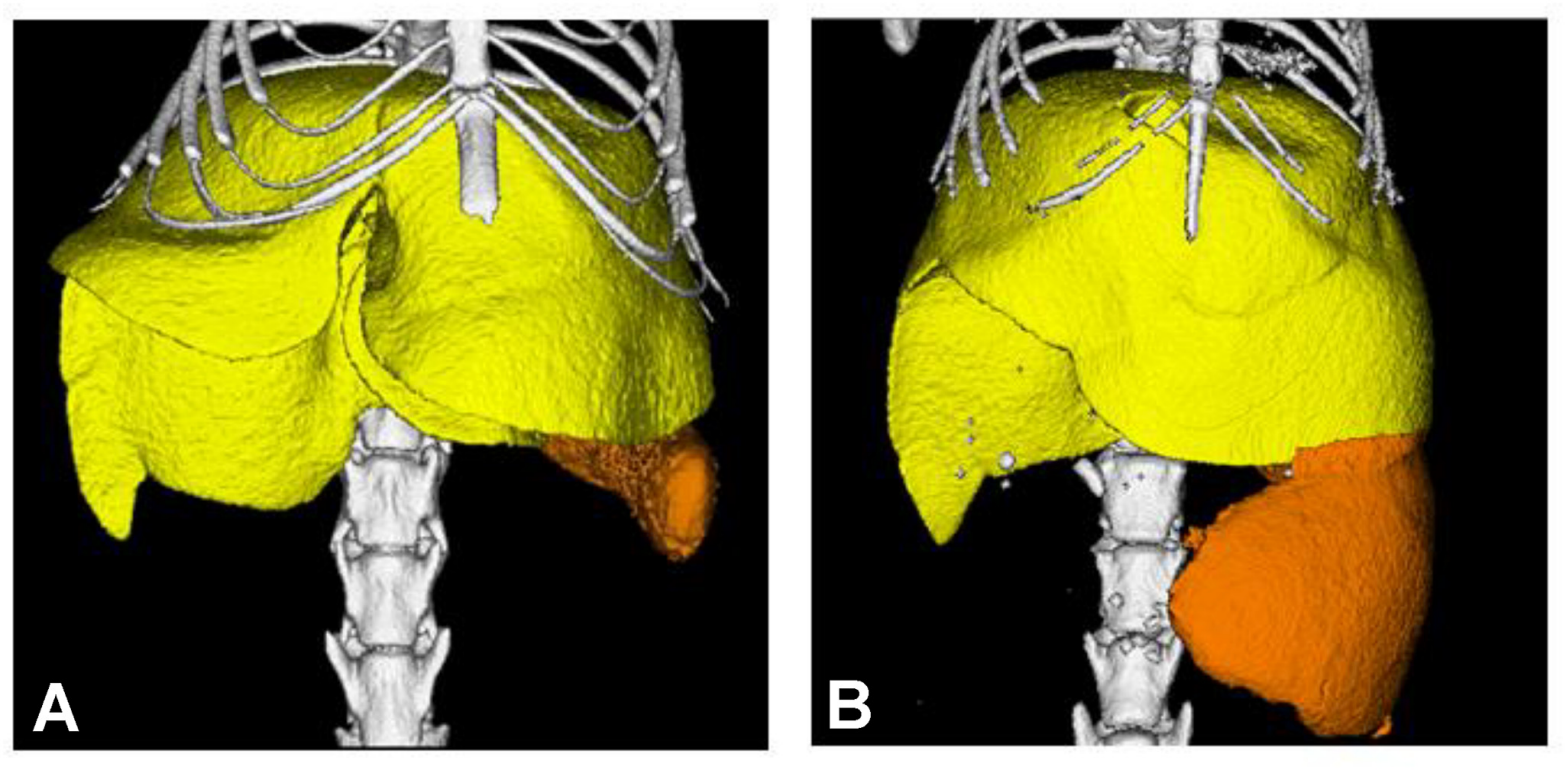
The emergence and/or progression of diseases may be monitored using μCT. In this case, μCT was used to visualize the increase in spleen volume in mice with bone cancer, 1 day after administration of contrast agent (FOV: 30mm×30mm; 2 min scan). (A) Mouse spleen before cancer agent injection. (B) Mouse spleen 5 weeks after cancer agent injection.

This procedure can be further applied towards the study of changes in organ size and morphology that correspond to the advancement of disease. For example, we were able to detect and quantify an enlargement in the spleen in the murine bone metastasis model, over the course of time (Fig 7 and S7 Movie). By leveraging the functionality of the contrast agent (its accumulation in tissue over time), we were able to observe the difference in spleen volume before and after cancer agent injection. Prior to the induction of cancer, the spleen volume was 76±14 mm^3^ (n = 2), whereas post-injection of the cancer agent, the spleen volume increased to 731±233 mm^3^ (n = 2).

## Discussion

Several factors, including image quality, long scan time, and x-ray dose have limited the application of *in vivo* micro-computed tomography in preclinical studies. In this study, we used a higher dosage of a known contrast agent (Exitron nano 12000) for preclinical *in vivo* micro-computed tomography of vasculature and organs at different hierarchical levels (anatomical landmarks) and time points in a murine bone metastasis model, with the goal of achieving improved image quality. We were able to validate that this pilot method, a combination of a powerful state of the art micro-CT machine, contrast agent and analysis software can be successfully employed to visualize and quantify vessels and organs *in vivo*, with imaging quality comparable to those obtained *ex vivo*. High quality images were generated with low scan time and at a low x-ray dose to each experimental animal. Utilizing this technique, we examined and monitored bone erosion and associated angiogenesis in the hind limbs of a murine bone cancer model. We also observed volumetric changes in the spleen in this cancer model.

Vessels and organs like the liver, kidneys and the spleen were imaged post contrast agent injection. We were able to demonstrate effective administration and toleration of the contrast agent in mice. A few previous studies encountered toxicity and mortality in the animals when higher doses were administered [12, 13]. Similarly, no changes in body weight or mobility were observed in the mice used for this study. Following contrast agent administration, we leveraged its differential accumulation, which allows for enhanced density contrast between soft tissues. As a result of the increased dose, improved visualization of organ boundaries and greater details of smaller vessels is made possible, facilitating enhanced segmentation of these structures than previously reported. Density maps and segmented views were generated of the murine liver, kidneys and heart, and their vasculature. The combination of organ and vascular segmentation together provides value to the understanding of disease propagation.

Prior studies on vascular and organ imaging in small animals primarily discuss lung, brain, cardiovascular, liver, and spleen imaging [5, 13, 18, 20–23]. These studies have not necessarily demonstrated adequate image quality for the monitoring of vascular changes associated with disease [10], rendering the visualization of smaller vessels in a disease model challenging [25]. In this study, several factors together helped to attain better image quality of smaller, thus far “evasive” vessels. The contrast agent Exitron nano provided high contrast of the soft tissues with a wide scanning time window (2-5h). High throughput, in terms of scanning speed (1–2 min) of the mice, was enabled by the Quantum *in vivo* CT, which also ensured a low x-ray dose exposure to the animals. And finally, the image analysis software (Analyze) was able to semi-automatically segment and quantify the soft tissues. As a result, we were able to visualize small vessels in a live mouse head, as well as segmented organs like the heart, liver and kidneys.

Small blood vessels are best quantified by corrosion casts or histological sections, but these techniques cannot be applied *in vivo* [25]. *In vivo* monitoring of angiogenesis with micro-CT provides better resolution and spatial information than MRI offers, especially when it comes to small blood vessels, although MRI is a traditional method of assessing in vivo angiogenesis. Amongst in vivo imaging modalities, micro—CT provides the highest resolution, and importantly, can be used noninvasively and longitudinally. CT is also relatively inexpensive compared to MRI, making it more easily available for use [33]. *In vivo* micro-computed tomography has been used to investigate tumor angiogenesis in small animals, and prior studies have detected between 30 and 50 μm diameters for tumor vessels using higher doses of iodine- or barium-containing contrast agents [10, 24]. Bone metastases are characterized by osteolysis and angiogenesis. Our *in vivo* images of cancer-induced bone destruction are comparable to prior in vivo microCT analysis of osteolysis [26, 27]. Angiogenesis in small animal models of bone metastases has been imaged using both *in vivo* and *ex vivo* microCT [4, 28], however the *in vivo* vascular imaging of the hind limbs has not been reported in normal or diseased mouse models, the animal of choice for experimental models of disease, due to advancements in mouse genomics [11, 34, 35]. In our study, we were able to visualize small vessels, down to 20 μm voxel size in the hind limbs of a murine bone metastatic model. Sufficient image quality enabled us to uniquely image bone and vasculature simultaneously *in vivo*. It should also be noted that our *in vivo* images of vasculature in the hind limbs are comparable to those produced by *ex vivo* microfil perfusion [4, 7], depicting clear connections of the vascular networks, and provides sufficient image quality for monitoring most other disease phenotypes.

We can visualize and monitor organ volume and morphology, as well as changes to these that are attributed to disease. In this way, deviations and/or abnormalities can be identified and quantified in other animal disease models. The study of changes to organ morphology corresponding to the advancement of disease would also be significantly abetted. It could also be used to evaluate tissue regeneration from tissue engineered implants [25]. Using this method, we were also able to detect and quantify spleen enlargement in this murine bone metastatic model, consistent with results from a prior study that reported the occurrence of tumor-related splenomegaly. It is believed that spleen enlargement occurs because of an accumulation of splenic granulocytes [36].

Preclinical *ex vivo* micro-computed tomography has been widely used for the structural assessment of tissues from animal disease models. It is capable of spatial resolution as low as 1 μm [13, 22]. *Ex vivo* micro-CT can produce high resolution images of microstructures, compared to *in vivo* (spatial resolution between 50–100 μm [10]), because it can overlook x-ray dose, contrast agent toxicity, and scan time [9], that *in vivo* imaging cannot. The nature of *ex vivo* imaging only allows for its use at the experimental endpoint. With *in vivo* imaging, researchers can noninvasively observe and study animal models prior to, in the course of, and following experimental intervention or drug administration, to quantitatively evaluate and monitor their effects at different time points, with comparable image quality to *ex vivo* [11]. For example, we tracked angiogenesis corresponding to bone erosion from 2 weeks to 5 weeks post cancer induction in our murine bone metastasis model.

In the future, a similar approach could be employed to monitor the *in vivo* efficacy of a therapeutic agent in treating the metastasis and associated angiogenesis. Additional image post-processing (i.e. ‘tortuosity’ index, color-coded Tree Analysis, etc.) and calculations (including measures of vascular density, average vessel radius/length for organs, etc.) could also be performed using more powerful software for further analysis of the chaotic vasculature at the bone metastasis site, compared to the contralateral knee, similar to what is done in a study by Vasquez et al. (2011) [37]. *In vivo* imaging, rather than *ex vivo*, could also be used to glean a great deal of information from various other animal disease models and mouse phenotype studies (i.e. Knock-out mice), if live tissue could be examined with strong contrast, as was in our case. At the same time, such non-invasive imaging would enable the use of fewer animals in studies, because the same animals could be imaged at different time-points, eliminating the need for sacrifices at multiple time points [12].

*In vivo* high-quality visualization of changes to vasculature and organ morphology offers dramatic advantages to the longitudinal monitoring and overall improved understanding of disease pathomechanisms, especially in devastatingly common maladies, like bone fracture healing or cancer [7, 18, 24, 25, 38]. Further, the *in vivo* quantification of hallmark disease manifestations, like the corresponding bone erosion and angiogenesis (within the same region) in our bone cancer model, is crucial [13, 39–41], and would deliver a sizeable impact to the further investigation, development and evaluation of novel drug candidates and therapies [2].

Small animal experiments necessitating repeated microCT scans and/or contrast agent injections must take into consideration the accumulation of contrast agent in the experimental animals. As mentioned earlier, the mice used in this study appeared to have tolerated the higher dose of the contrast agent well, with no direct, noticeable changes in their food or water intake, or movement. And, although the contrast agent would have been eliminated from the circulating bloodstream after a day [29], it would have accumulated in the macrophages, where it would remain for several weeks and months [21]. Following multiple injections of ExiTron nano 12000, a reduction in clearance rates was observed in mice [12]. Further investigation may be needed to determine the exact effects on the livers and spleens of mice that receive multiple injections of ExiTron nano, and whether any changes have occurred in the murine immune system, as a result. Identification of any long-term effects may alter the interpretation of results and/or outcomes. For studies that require imaging at multiple time points, additional options exist that can be considered, like pumps for the continuous administration of contrast agents [13], and use of the standard iodine-based contrast agents, which are low in toxicity [20]. These techniques could help address rapid bloodstream clearance, as well as any potential burdens to the livers and/or kidneys of the experimental animals.

Imaging at multiple time points would also require the effective minimization of x-ray dosage-related adverse effects to the experimental animals. In addition to being within the safety window of exposure, it is important to ensure that radiation doses do not introduce side effects or other deterministic effects that could lead to the misinterpretation of research results [42]. Based on scan parameters, the estimated radiation exposure is on the order of 85–746 mGy for each 2 min scan [43]. Within the literature, there is disagreement on what is a safe dose, depending on the type of tissue imaged [44, 45]. Bony structures absorb more radiation than soft tissue [46]. A similar local increase in dose would be expected in the contrast enhanced tissues after injection of a contrast agent. However, the scanning done in this study is well below the accumulated limit of while 5 Gy that has been reported to be a safe dose [45], and is also below the whole body lethal dose has been reported to be 5–7.6 Gy for mice [35, 47]. Radiation dose is also dependent on the strain of mouse, age at exposure, and other factors, including length of exposure, beam hardness, and desired image quality (resolution, noise, and contrast) [35, 46]. As the technology advances, new *in vivo* microCT systems, such as the PerkinElmer Quantum GX, and the Bruker 1278, enable quick and low-dose scanning that addresses radiation safety concerns in these small experimental animals.

## Conclusion

This pilot study was able to demonstrate that a higher dosage of ExiTron nano 12000 was well tolerated by mice, and offered higher vessel size resolution, down to 20 μm voxel size in a live hind limb murine bone metastasis model. A combination of this technique, the powerful micro-CT scanner and analysis software, enabled the visualization, segmentation, and quantification of various organs and their vasculature, including the liver, kidneys, and heart. It also allowed for the monitoring of morphological changes to vasculature and organ shape over time in the animal disease model. This technique can be applied to longitudinally monitor the progression of disease, especially cancer, to provide an improved understanding of its development, as well as aid in the evaluation of potential future drug treatments.

## Supporting Information

S1 Fig A Flowchart of the Image Processing Steps. (TIF)

S1 Movie Utilizing a high threshold (350 HU) to visualize and segment high density organs and vasculature in a live mouse. The main vessel and spleen have high density so they can be visualized and segmented using a high threshold.
(MOV)

S2 Movie Utilizing a low threshold (150 HU) to visualize and segment low density organs and vasculature. The liver and kidneys have low density, so they can be visualized and segmented using a low threshold.
(MOV)

S3 Movie Organ volume, morphology and vessel distribution visualized and segmented *in vivo*. The movie depicts the 3D rendering of specific organs within the mouse. Segmentation was performed sequentially and hierarchically—the main vessel, as well as contiguous networks of blood vessels, adjacent bone, and organs.
(MOV)

S4 Movie Semi-transparent 3D rendering of Organ volume, morphology and vessel distribution visualized and segmented *in vivo*. The movie depicts the 3D rendering of specific organs within the mouse, with the same sequential segmentation as in Fig 3 and S3 Movie. This movie also shows the overlay of the associated semi-transparent vasculature.
(MOV)

S5 Movie 3D volume-rendered movie of vasculature surrounding the mouse head. The superficial temporal vein and posterior facial vein can be viewed in great detail. Additional blood vessels that can be visualized include the inferior palpebral vein and anterior facial vein.
(MOV)

S6 Movie 3D volume-rendered movie of vasculature surrounding the mouse hip/hind limbs. The right and femoral arteries and caudal femoral artery can be seen as part of the network of blood vessels in the sacrum and hind limbs.
(MOV)

S7 Movie Spleen enlargement in a bone cancer mouse model. The movie depicts the enlargement of the spleen (segmented in brown) in a mouse with bone cancer in the right hind limb (as seen by the deterioration of bone in the right tibia and the associated angiogenesis).
(MOV)
