# Supplementary figures and images for: In Vivo Quantitative Microcomputed Tomographic Analysis of Vasculature and Organs in a Normal and Diseased Mouse Model
Source: PLoS One. 2016 Feb 24;11(2):e0150085. doi: 10.1371/journal.pone.0150085 (PMC4765930; doi:10.1371/journal.pone.0150085)

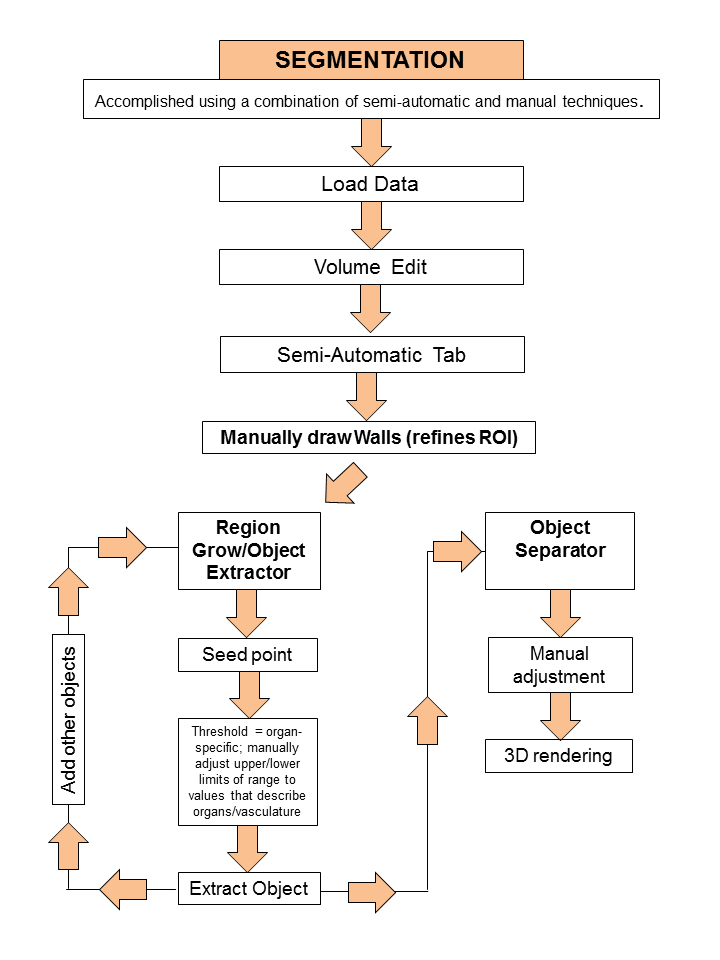

Supplement: S1 Fig — (TIF) [file pone.0150085.s001.tif]
